# Implementation and analysis of quantum computing application to Higgs boson reconstruction at the large Hadron Collider

**DOI:** 10.1038/s41598-021-01552-4

**Published:** 2021-11-24

**Authors:** Anthony Alexiades Armenakas, Oliver K. Baker

**Affiliations:** 1 Department of Physics, Harvard University, Cambridge, MA 02138 USA; 2 Department of Physics, Yale University, New Haven, CT 06520 USA

**Keywords:** Energy science and technology, Mathematics and computing, Information technology, Physics, Particle physics

## Abstract

With the advent of the High-Luminosity Large Hadron Collider (HL-LHC) era, high energy physics (HEP) event selection will require new approaches to rapidly and accurately analyze vast databases. The current study addresses the enormity of HEP databases in an unprecedented manner—a quantum search using Grover’s Algorithm (GA) on an unsorted database, ATLAS Open Data, from the ATLAS detector. A novel method to identify rare events at 13 TeV in CERN’s LHC using quantum computing (QC) is presented. As indicated by the Higgs boson decay channel $H\rightarrow ZZ^*\rightarrow 4l$, the detection of four leptons in one event may be used to reconstruct the Higgs boson and, more importantly, evince Higgs boson decay to some new phenomena, such as $H\rightarrow ZZ_d \rightarrow 4l$. Searching the dataset for collisions resulting in detection of four leptons using a Jupyter Notebook, a classical simulation of GA, and several quantum computers with multiple qubits, the current application was found to make the proper selection in the unsorted dataset. Quantum search efficacy was analyzed for the incoming HL-LHC by implementing the QC method on multiple classical simulators and IBM’s quantum computers with the IBM Qiskit Open Source Software. The current QC application provides a novel, high-efficiency alternative to classical database searches, demonstrating its potential utility as a rapid and increasingly accurate search method in HEP.

## Introduction

The search and selection of rare events in high energy physics (HEP) remains a significant challenge to modern scientific research, and new approaches are needed to rapidly and accurately analyze expanding databases. In the world’s largest particle accelerator, the Large Hadron Collider (LHC) at the CERN laboratory in Europe, accelerated particles are collided up to one billion times per second, generating a petabyte of collision data in the detectors each second. In these events, protons collide producing quarks, gluons, and rarely the Higgs boson, as the energies deposited in different layers of the detectors are recorded. Immense databases containing events recorded by the ATLAS (A Toroidal Lhc ApparatuS) detector must be filtered, sorted, and searched. The High-Luminosity LHC (HL-LHC) project, anticipated in 2026, will enable particle acceleration to unprecedented energies and proton-proton (*pp*) collision frequencies. Billions of particle events will be generated per second—approximately 5-to-7 times more data than currently produced—necessitating faster and more accurate analysis methods. The current study demonstrates a novel quantum computing (QC) method that identifies rare *pp* collision events in the LHC at 13 TeV energy.

Particle production events in the ATLAS detector are recorded as energy deposits in the calorimeter detector cells and as charged particle hits in the tracking detectors. Combinations of algorithms are used to make particle identifications and are analyzed using mainly classical algorithms run on classical computers. Computer algorithms assist by recognizing patterns of particle hits on the detector to identify and reconstruct individual particle trajectories. Distinguishing particle signatures from the background noise as they travel through the detector is the key to analyzing collision data, which has been time- and labor-intensive. The aim of the current study was to determine whether QC technologies and techniques may more efficiently select rare events from background as compared to classical computers. QC employs the quantum mechanical properties of quantum bits (qubits) to complete tasks with far greater efficiency than its classical computing counterpart. Prior to the current study, this fundamentally distinct form of computing had yet to be applied in the search of particle physics databases in this way.

Grover’s quantum algorithm^1,2^, a search algorithm designed to run on quantum computers, has been shown to search over a set of data quadratically faster than classical search algorithms. Grover’s algorithm (GA) selects a target quantum state and increases the probability that the system is measured in that state. In the current study, an application of GA was developed and applied to the search of an HEP database and run on a variety of quantum computers and simulators. The searched database from ATLAS Open Data comprises events from 13 TeV *pp* collision energy. The database contains data on lepton transverse momenta as detected by the ATLAS detector.

The Higgs boson that was discovered at the LHC^3,4^, if it is the standard model (SM) boson, will couple to SM particles in a manner that is unlike any other lepton, quark, or gauge boson; its coupling strength is related to the particle’s mass. If the nature of its coupling depends on the mass of the state it couples to, it may provide a new means to search for phenomena that are beyond the SM of particle physics. The Higgs boson could provide a portal to a so-called Dark Sector of new particles and interactions, coupling to them in a unique way that cannot be probed with other SM probes. Completely new physical states may therefore be accessible experimentally, via coupling to the Higgs boson, in a way that did not exist previously. Theoretical studies, supported by astrophysical and cosmological experimental data, indicate that these Dark Sector particles can lead to very rare events in LHC collisions^5–8^. An LHC Dark Sector search consists of detecting a resonance that decays to leptons. Leptons are a class of structureless particles with spin-1/2 that do not exhibit strong interactions. Leptons can be electrons (*e*), muons ($\mu$), tau particles ($\tau$), or neutrinos ($\nu _e$, $\nu _{\mu }$, $\nu _{\tau }$).

In applying GA to perform a quantum search on the database from the ATLAS detector for collisions’ products, or events, in which four leptons were detected at the appropriate energy and mass window, the presence of the Higgs boson in the post-collision particle showers was reconstructed, as indicated by the Higgs boson’s 4*l* decay channel. The analysis of this decay channel is a conduit for assessing Higgs boson decay to some new phenomena, such as $H\rightarrow ZZ_d \rightarrow 4l$. $Z_d$ refers to a hypothetical Dark Sector vector boson that is beyond the standard model of particle physics and is a focus of current research^9^. The study presented here demonstrates the development and application of GA to complete tasks and answer essential questions in particle physics—specifically, to search for signals of the Higgs boson decay products in events detected at CERN’s LHC, permitting reconstruction of the Higgs boson and demonstrating the utility of applying quantum computing to high energy particle physics.

## Results

GA was implemented to perform a quantum search on the database from the ATLAS detector for events in which four leptons were detected to target the presence of the Higgs boson in the post-collision particle showers, as indicated by the Higgs boson’s 4*l* decay channel (see Methods). The probability distribution of each quantum state’s measurement following steps and iterations of GA reflects the manipulation of amplitude to yield a peak in probability of target state measurement. The presented probability distribution results are obtained by repeating each quantum computation thousands of times on the initialized system and measuring the results after every run. Therefore, the probability distributions from measurement are determined with certainty. The divergence of experimentally obtained probability distributions *Q* from theoretically-calculated expected distributions *P* is used as a metric of performance, as described below and for each result. The Kullback–Leibler (K–L) divergence, denoted $D_{\text {KL}}\left( P \Vert Q \right)$, with logarithm base 2 was selected to quantify divergence and thus error. Each known distribution of states after running a quantum program on the system was calculated from state-vectors, which were mathematically computed. Results from the simulator diverge negligibly from corresponding known distributions due to the large number of samples. When analyzing results on quantum devices, the K–L divergence serves as a performance metric and quantifies decoherence, as explained in the following sections.

**Figure 1:**
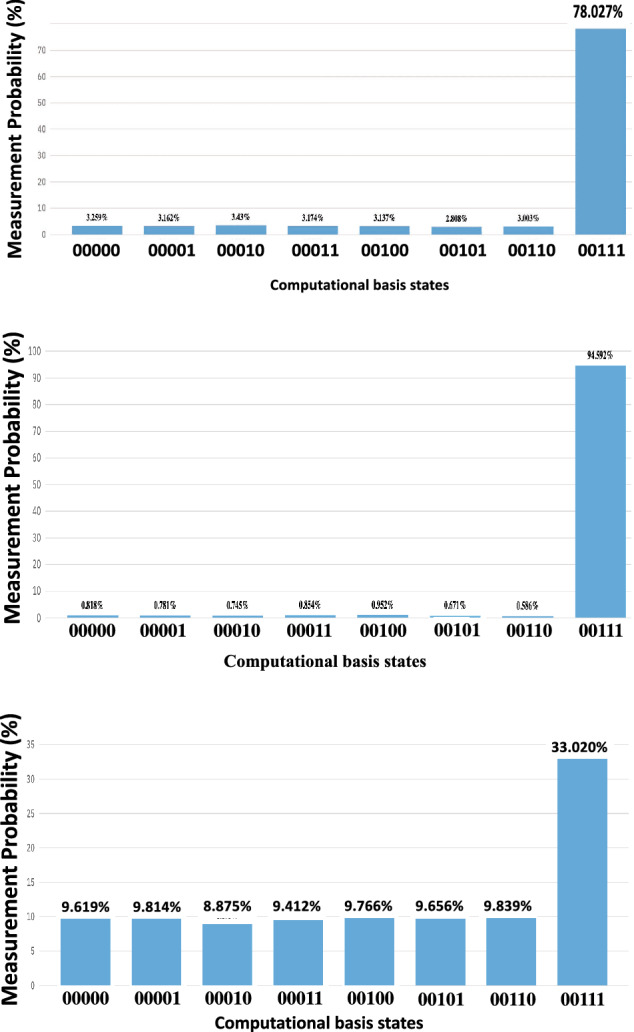
Probability of target state measurement with multiple iterations. The above results were obtained via sampling from simulated quantum state probability distributions after a varying number of GA iterations, repeated 8192 times for each number of GA iterations. The target state is $\mathinner {|{111}\rangle }$, denoted in the figure by $\mathinner {|{00111}\rangle }$. The number of samples establishes the accuracy of the presented probability distributions, which reflect probabilities defined by the state-vectors prior to measurement. Probability distributions obtained from simulations such as these, which sample from distributions defined by state vectors, negligibly diverge from these distributions when the number of samples is sufficient and can therefore be treated as accurate approximations. The approximation errors of the sampled distributions above, quantified by the Kullback–Leibler (K–L) divergence of the sampled distributions from the true distributions, are negligible ($5.36 \times 10^{-4}$ for one iteration, $8.54 \times 10^{-4}$ for two iterations, and $5.35 \times 10^{-4}$ for three iterations). In this work, K–L divergence is calculated with log base 2. Optimized probability of target state measurement occurs after $\frac{\pi }{4}\sqrt{2^n}$ iterations, rounded to the nearest integer. For a three-qubit quantum system the optimal number of iterations is $\frac{\pi }{4}\sqrt{2^3} \approx 2.22 \approx 2$, matching the results.

A three-qubit quantum chip placed in an equal superposition demonstrates a 1/8 probability of each possible state being measured. After one iteration of GA, there was a probability of 0.78 that the chip was measured in the target state (Fig. 1). Following two iterations of GA on three qubits, the target state was selected with a probability of 0.94 on the QC simulator. Our results shown in Fig. 1 corroborated models for the optimization of GA iterations, which indicate that the ideal number of iterations on n qubits is $\frac{\pi }{4}\sqrt{2^n}$ assuming no hardware error^10^. When three iterations of GA were run on a three-qubit system, the correct state was selected with a probability of 0.33. Figure 1 shows the results of GA with a varying number of iterations on three qubits. The cited probabilities of measurement in any state were obtained by measuring 8192 quantum systems after one, two, and three GA iterations and computing the percentage of measurements which recorded the state. The optimization of target state measurement probability is a key element of conducting quantum searches with GA; this factor was considered along with quantum decoherence when applying the quantum search to ATLAS data.

**Figure 2:**
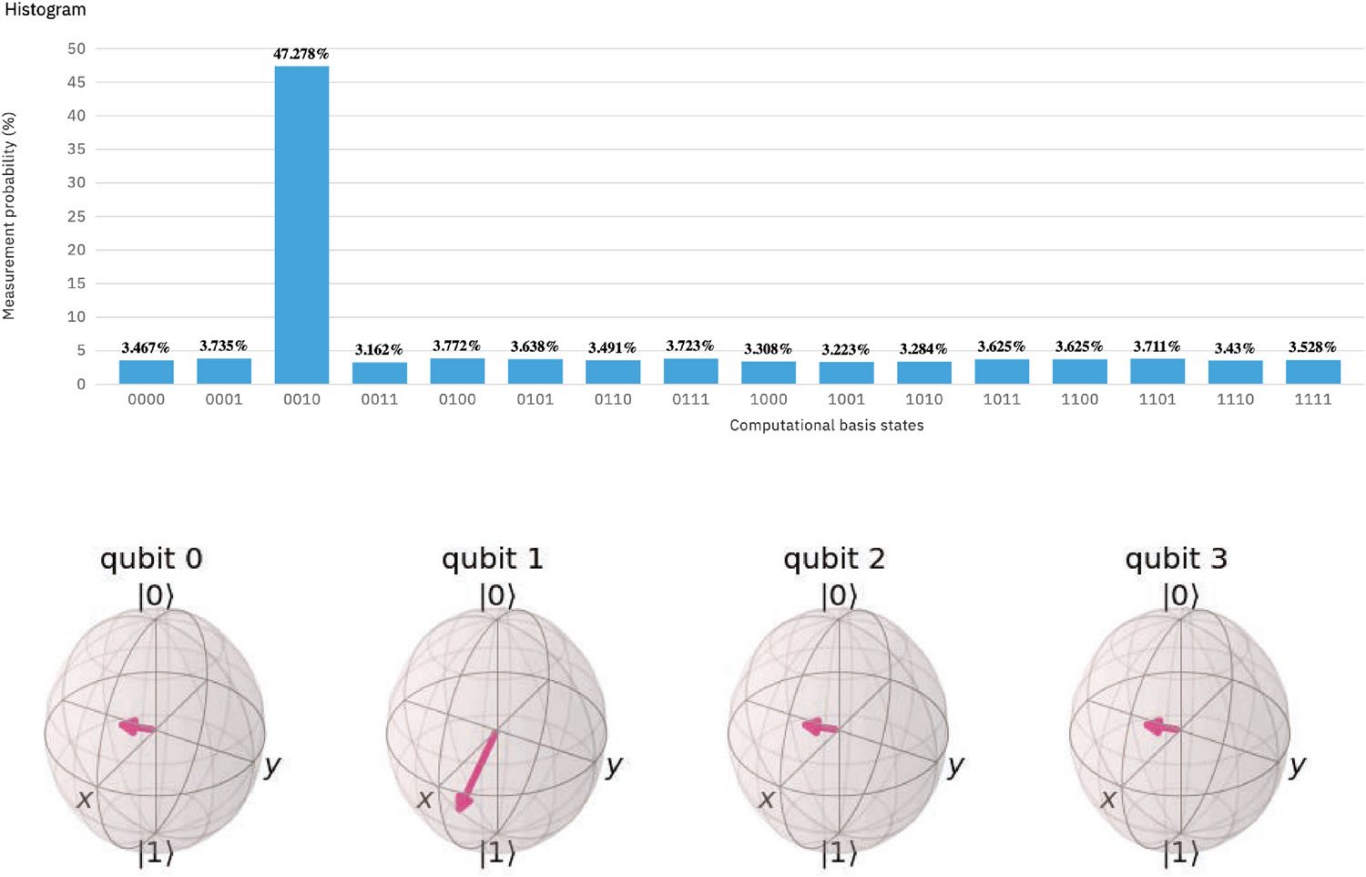
Four-qubit quantum search and qubit representation. The algorithm yielded a distinct peak in probability of target state measurement (left) when run on four qubits. The probability distribution is an approximation of the state-vector-defined probability distribution via sampling, with 8192 samples, and therefore has a negligible K–L divergence (${1.15\times 10^{-3}}$) from the known distribution. The final multi-qubit state that is conducive for a successful search is shown in Bloch Sphere representations of each qubit (right).

GA was run on a varying number of qubits. The algorithm continued to yield a distinct peak in target probability after one iteration, as seen in Fig. 2. Yet, as the number of qubits and thus possible states increased, correct measurement was made with a lesser probability. Figure 2A shows a histogram of measurements made after one iteration of GA on four qubits. As shown, one iteration of GA left the four-qubit system with a 0.47 probability of correct measurement, which is less than the aforementioned probability of correct measurement after one iteration on three qubits as shown in Fig. 1. As $\frac{\pi }{4}\sqrt{2^n}$ GA iterations optimize selection accuracy, an increasing number of iterations is needed to sufficiently raise target state probability as the number of qubits increases. However, when run on real devices, longer quantum codes fall prey to decoherence, and iteration number is thus best kept to a minimum for a more effective quantum search.

**Figure 3:**
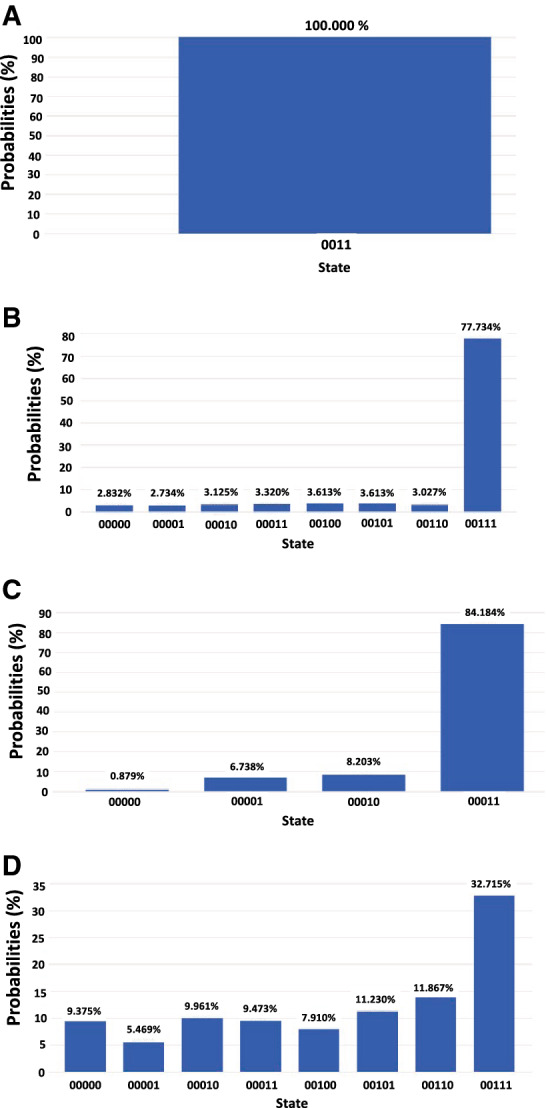
Grover’s algorithm applied to two- and three-qubit systems in Qiskit. The probability of each two-qubit (**A, C**) and three-qubit (**B, D**) state is presented upon measurement after one iteration of GA on the Qiskit simulator (**A, B**) and on real IBM QCs (C, D). The K–L divergence of real QC results from simulated results is 0.248 for two qubits and 0.603 for three qubits.

GA was executed on increasing numbers of qubits on real IBM quantum computers, which demonstrated a similar peak distinction once measured. The probability distributions of runs on QCs were compared to those of simulated runs. As seen in Fig. 3, the peak in probability was slightly less distinct after runs on real QCs due to quantum decoherence. The three-qubit distribution obtained from real QC experiments had a K–L divergence of 0.615, greater than the K–L divergence of the two-qubit state distribution from its corresponding expected distribution, which was 0.248.

### Quantum search of LHC open data

#### First encoding

**Figure 4:**
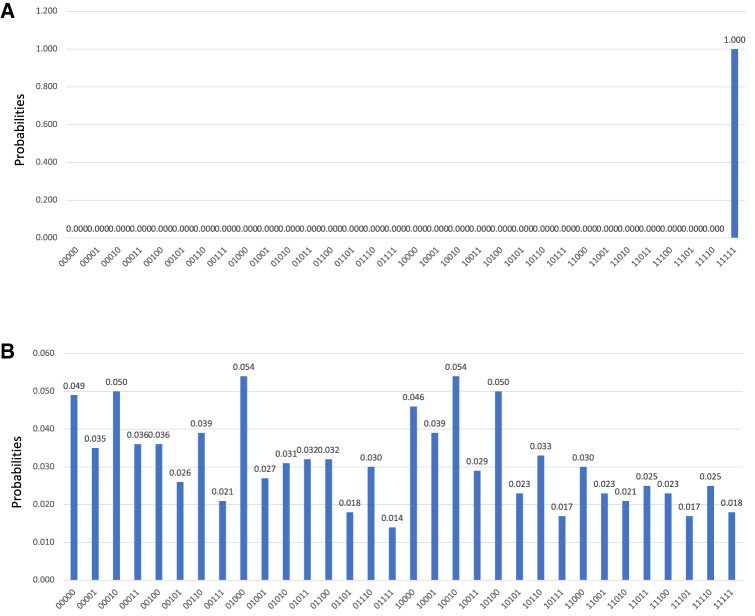
Histogram of probabilities by quantum state with the ATLAS data encoded. (left): The group of four data entries included the target lepton index = 3. The algorithm was run on Qiskit’s ibmq_qasm_simulator backend, a classical simulation. B (right): This group of four data entries contained the target lepton index = 3. The effects of decoherence can be seen. The K–L divergence of the experimental distribution from the simulated distribution is 5.79 The algorithm was run on the quantum device ibmq_16_melbourne.

**Figure 5:**
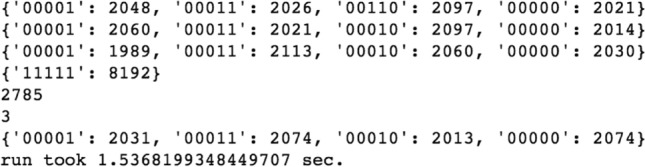
Final state measurement counts for five groups of four data entries, each searched by GA. The fourth group contains the target lepton index = 3, and its corresponding state was measured all 8192 times on the simulator backend.

**Figure 6:**
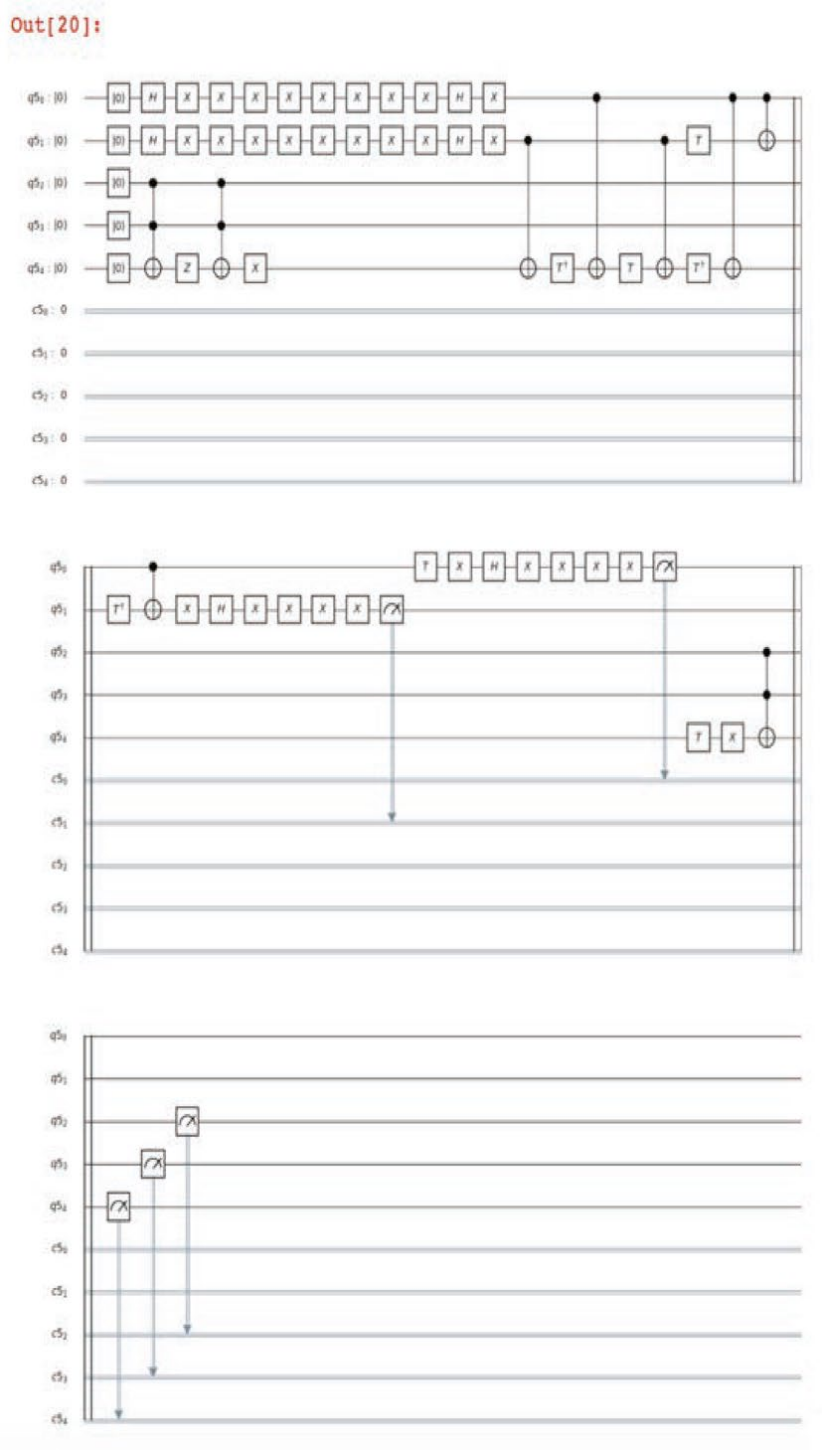
Quantum circuit. A quantum circuit generated in the selection process.

The classical simulator backend ibmq_qasm_simulator yielded the target state with a probability of 1.0 (100%), as seen in Figs. 4 and 5. The search run on the real 15-qubit quantum computer ibmq_16_melbourne demonstrated a limited peak amplification, with the distribution’s K–L divergence from the simulated results equal to 5.79, the consequence of quantum decoherence (Fig. 4). The code that was written generates distinct quantum circuits depending upon the values in the database. One quantum circuit, a diagram of all the quantum gates implemented in one run of Grover’s algorithm on the ATLAS data, is shown in Fig. 6.

#### Second encoding

**Figure 7:**
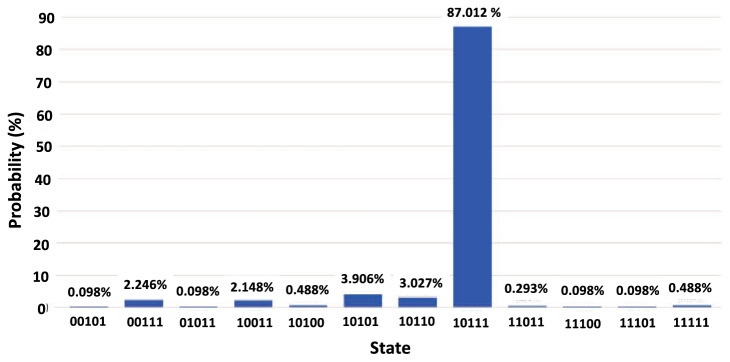
Histogram of final state probabilities after 8192 runs on the quantum computer ibmq_vigo. The correct state was selected with a probability of 87.012%, or 7128 of 8192 runs. The 20 of 32 states not shown in the histogram were states in which the system was not measured on any run. The K–L divergence of this distribution from its corresponding simulation is 0.475.

The search using the second encoding method was run on QCs and QC simulators, all obtaining results that reflect success. As with the first encoding method, the simulator selected the correct state with a probability of 1.0, or 100%. On the quantum computer ibmq_vigo, the five-qubit system was measured in the correct state 87.0% of the time, as seen in Fig. 7. For these results, $D_{\text {KL}}\left( P \Vert Q \right) = 0.475$, significantly reduced as compared to the first method. With limited quantum decoherence owing to the modified, more efficient encoding method, the runs of the real quantum computer were successful.

## Discussion

We describe the development and implementation of a novel quantum search method to identify rare particle collision events in ATLAS Open Data using GA on several quantum simulators and quantum computers. The latest ATLAS Open Data release contains databases of *pp* collision events at a highest-ever 13 TeV collision energy, and the current QC application to data from *pp* collisions at 13 TeV collision energy is novel. The 100% correct selection rate when GA was run on simulated qubits in the R programming language and in IBM’s Qiskit using Python on QC simulators demonstrated here indicates that, mathematically, the maneuvers on qubit amplitudes successfully yielded the correct result upon measurement.

**Figure 8:**
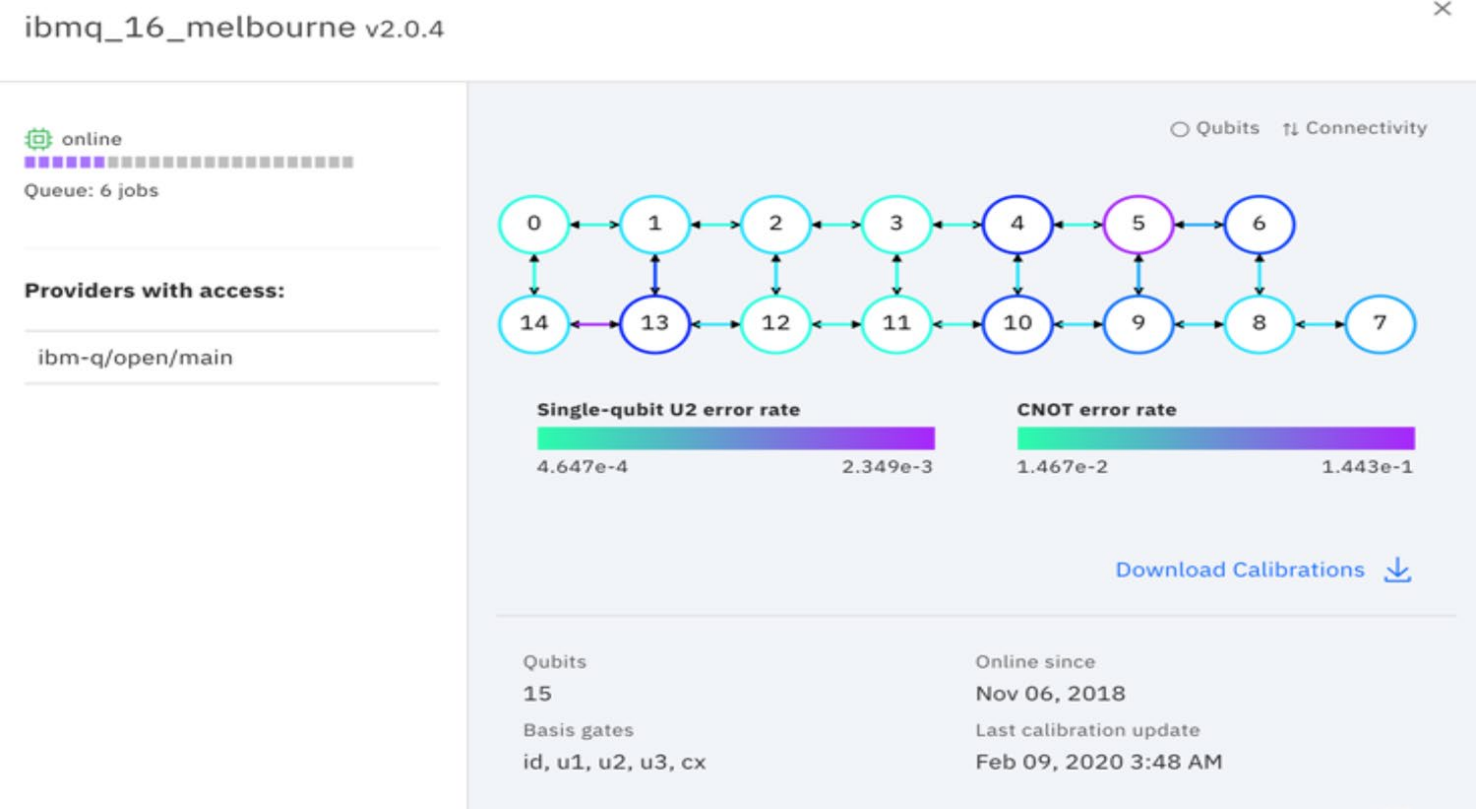
A diagram of single- and multi-qubit error rates in ibmq_16_melbourne, provided by IBMQ.

When the first encoding method was run on the 15-qubit backend ibmq_16_melbourne, where the quantum speed-up is reaped, the peak amplification was reduced as compared to the simulator. The reduction in amplification was a product of quantum decoherence, the interaction of qubits with their environment that randomizes results in QC, as seen in Fig. 4. We examined ways to reduce the noise in our results. A comparison of K–L divergences corroborates that longer quantum codes generally cause a system to lose coherence more so than shorter ones. The K–L divergence of real result distributions from simulated ones was greater on three qubits (0.603) than on two (0.248). The three-qubit system, which requires a longer series of gates than the two-qubit system to perform an iteration of GA, diverges from simulation more so than the two-qubit system does. Accordingly, with the reduction of quantum gates in our code, the effects of decoherence were mitigated. Single qubit (U2) and inter-qubit (CNOT) error rates are shown in Fig. 8. While relatively low for each gate, the error rates compound over the quantum circuit. The more gates used in a quantum code, the greater the compound error rate becomes, and the greater the likelihood that qubits interact adversely with their environment. The effects of decoherence were quantified by the K–L divergence, with a greater K–L divergence corresponding to more decoherence and poorer performance. Current research efforts are directed toward combating decoherence. The improving engineering of quantum computers offers promise in limiting error in the execution of longer quantum codes.

A second encoding method was developed to decrease the number of gates and thus the compounded error rate of the quantum search. The resulting decrease in discrepancy between the results of the QC simulator and those of the real quantum devices is a reflection of mitigated error. In a simulated environment, without quantum decoherence, GA successfully and effectively searched ATLAS Open Data for events with four leptons—with 100% accuracy. GA selected the target state with high probability when run on a real QC, demonstrating the viability of this advantageous search method. The reduced K–L divergence of the real QC result from the result on the simulator, from 5.79 to 0.475, indicates the reduction of decoherence with the second encoding. The presented results are promising in databases directly formatted to be searched by quantum computers; with LHC databases constructed in this way, quantum computers will expedite the data sorting, search, and analysis processes.

The HL-LHC will produce increased event rates and a more challenging environment for selection of rare signal events of interest to scientists against a large bevy of scientifically inconsequential background. Selection of rare, interesting events as described in this article requires algorithms that can select and then format the detector output into a form that can be recognized and used by quantum algorithms, such as GA. We have demonstrated one method as an example—GA applied to properly formatted Higgs boson events in the ATLAS collaboration. The data was converted from formats conventionally used in classical analysis to quantum formats that are used by a quantum algorithm. The selection of events with four leptons, essential in identifying the Higgs boson decay channel $H\rightarrow ZZ^*\rightarrow 4l$, may be used to assess Higgs boson decay to some new phenomena, such as $H\rightarrow ZZ_d \rightarrow 4l$. GA applied to the database of events described here demonstrates a way forward for use with varied databases composed of unsorted particle physics data events.

## Conclusion

The search presented tackled the enormity of HEP databases in an unprecedented manner, identifying and reconstructing events at 13 TeV on a quantum computer. The 100% success rate of Grover’s algorithm in the simulated environment in searching the ATLAS Open Data for four leptons in the same event substantiates the theoretical viability of GA. The algorithm’s success on quantum computing simulators extends to the runs on real quantum computers, wherein the benefits of quantum computing to efficiency lie. Our findings indicate that the use of quantum computing in particle physics would contribute to the acceleration of scientific discovery by, as shown via both the simulation as well as quantum computers, improving data selection at the LHC. With the anticipated LHC upgrade and database expansion, the current results offer a novel approach to more efficiently conduct searches for rare events in high energy physics. The outcome is a valuable set of results that offer promise to both quantum computing and particle physics, and to the application of the latter to the former.

## Methods

### Higgs boson search formulation

The search was conducted for a resonance that decays to electron-positron or positive muon and negative muon pairs. This resonance that signals a new particle would be seen above a broad spectrum of background SM events. The off-shell decays are broad distributions in mass and do not produce a narrow resonance. The resonance that signals a new particle is a narrow peak on top of the broad, featureless distribution. Electron and positron candidates consist of clusters of energy deposited in the electromagnetic calorimeter associated with their tracks inside the detectors. The clusters matched to tracks are required to satisfy a set of identification criteria that require the longitudinal and transverse shower profiles to be consistent with those expected for electromagnetic showers. These are registered as hits, which are then translated in software to electron and positron energies, momenta, charges, and position at any given time.

Positive and negative muon candidates are formed by matching reconstructed detector tracks in one ATLAS spectrometer subsystem with either complete or partial tracks reconstructed in a separate ATLAS subsystem. If a complete track is present, the two independent momentum measurements are combined. The particles are identified as muons if their calorimetric energy deposits are consistent with a minimum ionizing particle.

In this current analysis, the detector data had already been formatted to run using classical algorithms on a classical computer. This format was changed to suit the quantum algorithm (GA) that is being used for the results shown here. It is understood that for future analyses that will employ quantum search algorithms such as the one described here, it will be much more efficient to store that data in a quantum format initially. This formatting should be the next step taken after collecting the tracking hits, energy deposits, shower profiles, etc, as described above.

The collected information, as described in the previous paragraphs, was converted into database entries that are appropriate for the designed method of Dark Sector searches using exotic decays of the Higgs boson, and which are in a format that can be understood and handled by the quantum search algorithm. In this study, Grover’s quantum search algorithm was used, and the database searched contained the transverse momenta of detected leptons.

Data pertaining to the exotic decays of the Higgs boson used in Dark Sector searches was sought. The Higgs boson may decay into a $ZZ^*$ ($Z^0$ boson and an off-shell $Z^0$ boson) pair, which in turn decay into four leptons. This decay channel is commonly represented $H \rightarrow ZZ^* \rightarrow 4l$^3,4^. $H \rightarrow ZZ_d \rightarrow 4l$, where the $Z_d$ refers to a Dark Sector vector boson that is beyond the standard model of particle physics, can occur^9^. Once produced, the Higgs boson decays quickly, leaving the four leptons at the end of the decay channel to be detected by the ATLAS detector. A search was designed on a database of detected leptons; the search was for collisions, or events, in which four leptons were detected. The data analyzed via quantum computing for this existing work was taken from the LHC’s recent run at a record-high proton-proton collision energy of 13 TeV, where the chances of Higgs boson production are higher.

### Grover’s algorithm and implementation

#### Classical search algorithm

Grover’s algorithm was implemented in the search process on real data. Before it was used to develop a search of real ATLAS data, GA was run on the qubits of several quantum computing simulators and real quantum computers. GA searches a database of N entries, where N is some integer. A classical computer (composed of bits, as opposed to qubits), goes through each entry until the target entry is found.

On average, the computer must make (N+1)/2 probes before the target entry is selected, assuming an equal probability that each entry is the target entry. To demonstrate that on average (N+1)/2 probes are made on a classical computer, take a list of N database entries, where each can be the target entry. For target entry *i* in {1, N}, the classical computer probes each entry in {1, *i*} before making the correct selection; thus it takes *i* probes to reach the correct selection of the target entry *i*. The sum of all probes for all potential target entries is $\sum \limits _{i=1}^N i = \frac{N(N+1)}{2}$, and the number of potential searches is N, so the average number of probes per search is $\frac{\frac{N(N+1)}{2}}{N} = \frac{N+1}{2}$.

#### Quantum search algorithm

Quantum search algorithms execute the search in a fundamentally different manner and exhibit a quadratic speedup over classical algorithms. On a two-qubit quantum computer, there are four measurable states, as each qubit can be measured in the $\mathinner {|{0}\rangle }$ or $\mathinner {|{1}\rangle }$ state (yet may exist in a superposition of states prior to measurement). On a three-qubit quantum computer, there are 8 measurable states ($\mathinner {|{000}\rangle }$, $\mathinner {|{001}\rangle }$, $\mathinner {|{010}\rangle }$, $\mathinner {|{011}\rangle }$, $\mathinner {|{100}\rangle }$, $\mathinner {|{101}\rangle }$, $\mathinner {|{110}\rangle }$, and $\mathinner {|{111}\rangle }$). Thus in general, on an n-qubit quantum computer there are 2$^n$ measurable states. In quantum superposition, each state has an amplitude, such that a three-qubit state in superposition can be written as:

1 $$\begin{aligned} \mathinner {|{\Psi }\rangle } = a\mathinner {|{000}\rangle } + b\mathinner {|{001}\rangle } + c\mathinner {|{010}\rangle } + d\mathinner {|{011}\rangle } + e\mathinner {|{100}\rangle } + f\mathinner {|{101}\rangle } + g\mathinner {|{110}\rangle } + h\mathinner {|{111}\rangle } \end{aligned}$$

where a, b, c, d, e, f, g, and h are the amplitudes of each measurable state in superposition. The modulus squared of the amplitude of a state is the probability of its measurement in that state. The sum of the probabilities of each state’s measurement is 1. Thus,

2 $$\begin{aligned} {|a |^2} + {|b |^2} + {|c |^2} + {|d |^2} + {|e |^2} + {|f |^2} + {|g |^2} + {|h |^2 }= 1. \end{aligned}$$

**Figure 9:**
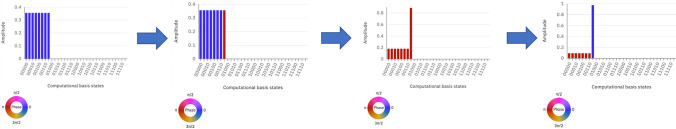
The effect of Grover’s algorithm on the the amplitude of each qubit state. The amplitudes of all 8 possible states of the three-qubit quantum chip are shown at various stages of the algorithm. The system is placed in an equal superposition (far left), with each state’s amplitude being $\frac{1}{2\sqrt{2}}$. Progressing to the right, the phase transformation and amplitude amplification of the target state are shown. The process is repeated in a second iteration, resulting in a greater target state amplitude (far right).

GA searches for a target state by amplifying the state’s amplitude, minimizing the amplitudes of all other states. The end result is that the qubit system has a higher probability of being measured in the target state^11^. The first step of GA is the placement of all states in an equal superposition, where their amplitudes are equal. Then, GA manipulates the amplitudes of the qubit system, selecting the target state’s amplitude and amplifying it in what is called the “Grover iteration”^12^. Figure 9 demonstrates how the Grover iteration alters measurement probability. The three-qubit quantum chip is placed in an equal superposition, with each state’s amplitude being $\frac{1}{2\sqrt{2}}$. The target amplitude is multiplied by −1 (a phase shift of $\pi$) before the algorithm performs amplitude amplification on the target state. After the second GA iteration, the target state amplitude, and thus the state’s probability of measurement, is closer to 1. Quantum algorithms that do not achieve a 100% success rate sacrifice accuracy for speed over classical algorithms, which perform selection with certainty. However, due to the greater computational complexity and thus slower execution of classical searches as illustrated above, searches on large databases such as the one in the study become impractical and less effective with classical algorithms.

### Computing known probabilities

The effects of the Grover iteration on quantum state-vectors can be computed via matrix operations and used to measure the success of algorithm runs. QC simulators compute exact known probabilities via state-vectors and sample from those determined probabilities to obtain distributions over quantum states. GA first marks the target state from equal superposition by performing a phase change of $\pi$, effectively multiplying the target state amplitude by $-1$. Then, the amplitudes are reflected about their mean value. By representing the gates that perform the marking and amplification as unitary transformations of state-vectors, the final states of the system after GA are determined. Since each state amplitude is related to the probability of measurement in that state, the expected probability distribution can thus be computed. During one iteration of GA on a three-qubit system with marked state $\mathinner {|{111}\rangle }$, the state of the system is transformed from equal superposition $\mathinner {|{\Psi _H}\rangle }$ to $\mathinner {|{\Psi _R}\rangle }$ via transformation of target state amplitude, and finally to $\mathinner {|{\Psi _f}\rangle }$ via amplitude amplification, as per the equations:

$$\begin{aligned} \mathinner {|{\Psi _H}\rangle }&= \frac{1}{2\sqrt{2}}\mathinner {|{000}\rangle } + \frac{1}{2\sqrt{2}}\mathinner {|{001}\rangle } + \frac{1}{2\sqrt{2}}\mathinner {|{010}\rangle } + \frac{1}{2\sqrt{2}}\mathinner {|{011}\rangle } + \frac{1}{2\sqrt{2}}\mathinner {|{100}\rangle } + \frac{1}{2\sqrt{2}}\mathinner {|{101}\rangle } \\&\quad + \frac{1}{2\sqrt{2}}\mathinner {|{110}\rangle } + \frac{1}{2\sqrt{2}}\mathinner {|{111}\rangle } \\ \mathinner {|{\Psi _R}\rangle }&= \frac{1}{2\sqrt{2}}\mathinner {|{000}\rangle } + \frac{1}{2\sqrt{2}}\mathinner {|{001}\rangle } + \frac{1}{2\sqrt{2}}\mathinner {|{010}\rangle } + \frac{1}{2\sqrt{2}}\mathinner {|{011}\rangle } + \frac{1}{2\sqrt{2}}\mathinner {|{100}\rangle } + \frac{1}{2\sqrt{2}}\mathinner {|{101}\rangle } \\&\quad + \frac{1}{2\sqrt{2}}\mathinner {|{110}\rangle } - \frac{1}{2\sqrt{2}}\mathinner {|{111}\rangle } \\ \mathinner {|{\Psi _f}\rangle }&= \frac{1}{4\sqrt{2}}\mathinner {|{000}\rangle } + \frac{1}{4\sqrt{2}}\mathinner {|{001}\rangle } + \frac{1}{4\sqrt{2}}\mathinner {|{010}\rangle } + \frac{1}{4\sqrt{2}}\mathinner {|{011}\rangle } + \frac{1}{4\sqrt{2}}\mathinner {|{100}\rangle } + \frac{1}{4\sqrt{2}}\mathinner {|{101}\rangle } \\&\quad + \frac{1}{4\sqrt{2}}\mathinner {|{110}\rangle } + \frac{5}{4\sqrt{2}}\mathinner {|{111}\rangle } \end{aligned}$$

The measurement probability of each of the eight basis states in $\mathinner {|{\Psi _f}\rangle }$ defines a distribution $P = ({\frac{1}{32}, \frac{1}{32}, \frac{1}{32}, \frac{1}{32}, \frac{1}{32}, \frac{1}{32}, \frac{1}{32}, \frac{25}{32}})$ where *P*(*i*) is the probability that state $\mathinner {|{i}\rangle }$, with *i* represented in binary, is measured.

This process was repeated to generate distributions for two- and four-qubit systems after one GA iteration, as well as for three qubits after two and three GA iterations.

### Quantifying result success with K–L divergence

After calculating probability distributions directly via the quantum states, these distributions were used as benchmarks against which algorithm performance was assessed. The metric of performance was the Kullback–Leibler (K–L) divergence $D_{\text {KL}}\left( P \Vert Q \right)$ of experimental distributions *Q* from their corresponding known distributions *P*. The K–L divergence is defined by:

3 $$\begin{aligned} D_{\text {KL}}\left( P \Vert Q \right) = \sum _{i} P(x_i) \log _{2} \frac{P(x_i)}{Q(x_i)} \end{aligned}$$

This method was used to compare the performance of quantum computers to quantum simulators, as $D_{\text {KL}}\left( P \Vert Q \right)$ is a measure of difference between two probability distributions, *P* and *Q*, and can be thought of as the information lost when replacing *P* with *Q*. $D_{\text {KL}}$ increases as the difference between distributions increases; therefore, the more a quantum system experiences decoherence and measurements deviate from the expected probability distribution, the greater the $D_{\text {KL}}$. Analysis of results using the K–L divergence allowed for a concrete comparison of methods and a definitive assessment of results.

### Quantum search algorithm application

GA was first run in R Programming’s quantum computing simulator. The QC simulator in R simulates a quantum computer using matrices and samples from a distribution of state measurement probabilities during the measurement step. In place of physical changes of qubit amplitudes, the simulator runs mathematical transformations.

Registration with IBM granted access to real quantum computers in addition to QC simulators through Qiskit. From then on, a Jupyter Notebook was employed to create and modify code in Python. The simulation backend ibmq_qasm_simulator was used for testing numerous times before algorithms were run on real devices. After running GA in different ways on both real and simulated quantum hardware as to develop the best method for application to real encoded data, the search of LHC data was developed.

#### ATLAS database search formulation

**Figure 10:**
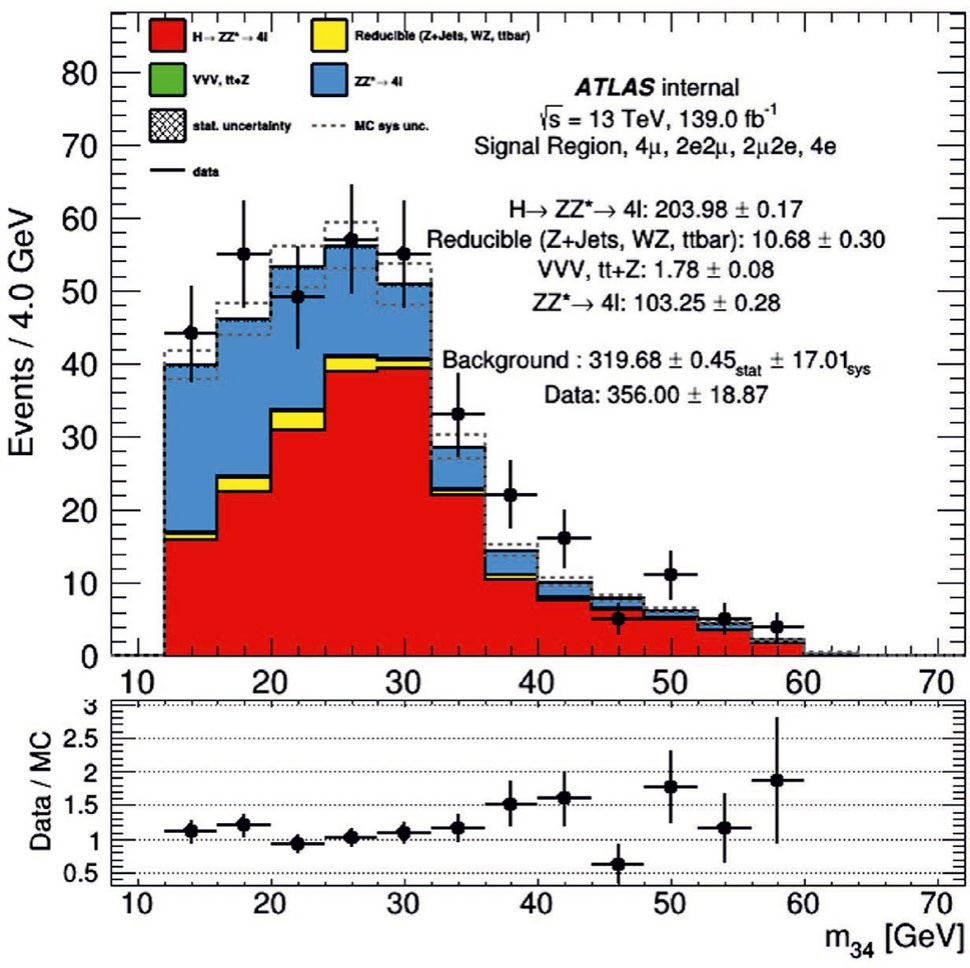
Original Data Distribution The distribution of $H\rightarrow ZZ^*\rightarrow 4l$ decay data as detected by the ATLAS collaboration.

The database contained the transverse momenta (the component of momentum that is perpendicular to the beam line of collided particles) of detected leptons. Leptons were recorded with their event number. To distinguish between leptons in the same event, “instance” values were assigned in the ATLAS database. Any first lepton detected in its event had an instance = 0; the second detected lepton in its event, an instance = 1; for the third, instance = 2, and for the fourth, instance = 3. Thus, the presence of a lepton instance value of 3 in the database indicated that four leptons were detected following one collision. Therefore the quantum search was formulated to find the $H \rightarrow ZZ^* \rightarrow 4l$ and $H \rightarrow ZZ_d \rightarrow 4l$ signal processes by searching for lepton instance values of 3. There are more than nine dozen variables (features) in a typical data set of four-lepton final states after proton-proton collisions. The analysis of four-lepton data selected via quantum computing as described in this document is concentrated on the event (collision) number, the number of leptons in (produced by) that event, and the transverse momenta (in units of GeV/c) of the leptons in the event. Figure 10 is a distribution of ATLAS data used in this study.

In order to apply the quantum search algorithm to the ATLAS data, the data was encoded into the quantum circuit. Classical data, in groups of four entries, was placed into the quantum states of a five-qubit quantum system as follows. As each lepton received an “instance” value for the event in which it was detected by ATLAS, the marked state corresponded to instance = 3. The search was run on the five-qubit quantum system, with q[0] and q[1] defining the index of the lepton in its group of four leptons searched by GA, and with q[2] and q[3] keying in the value of the lepton “instance” with respect to event, as in the database. Qubit q[4] served as an ancillary qubit. A Jupyter Notebook was used to compose code to run on IBM devices.

Our code was run on each group of data entries thousands of times to yield accurate probability distributions for the final state of the quantum system. All iterations on the data subsets not containing the marked lepton state yielded a quantum system in equal superposition and thus an equal probability of measurement in each state. Those iterations on groups of four data entries containing an index value of 3 were hypothesized to leave the quantum system with a higher probability—mathematically, 100%—of measuring the marked state with index = 3. In order to solely yield the data entry (row) number in the database and the transverse momentum of exclusively leptons with index = 3, the code yielded these values for those states with a probability of being measured $\ge$ (.80). The algorithm was first run and tested on IBM’s ibmq_qasm_simulator backend before it was run on several IBM quantum computers.

#### Second ATLAS database search formulation

After decreasing the number of quantum gates and running the code on several devices with the lowest error rates, we concluded that with the current level of QC engineering, decoherence to the degree of limiting meaningful results occurred with the amount of quantum gates our primary method of encoding the data requires.

A different method of encoding data into the quantum computer was developed for the purpose of obtaining successful results with the latest existing quantum computers rather than anticipating advancements in their engineering. We sought a method successful beyond theoretical quantum information. Our second method of encoding classical data to be searched requires fewer quantum gates and saw successful results on both the simulator and real quantum computers.

The casting of unsorted data—in groups of 8—into the quantum computer began by defining qubits q[0] and q[1] as the value register. These two qubits encode the instance values of the database, which, as previously, are in the set {0,1,2,3}, as did q[2] and q[3] in the primary encoding method already described. In this second method, q[2], q[3], and q[4] served to facilitate a binary encoding of index values contained in the set {0,1,...,7}.

The result is the amplification of a state denoted by a five-digit binary value with the first three digits corresponding to the specific index value of the 4-lepton event and the last two representing the instance value. The final measured state can thus be read as $\mathinner {|{vwxyz}\rangle }$ where $\mathinner {|{vwx}\rangle }$ is the measured three-qubit state of q[4], q[3], and q[2] in that order, and $\mathinner {|{yz}\rangle }$ is the measured two-qubit state of q[1] and q[0]. Both *vwx* and *yz* are series of 0’s and 1’s, where *yz* represents the searched database value in binary, and *vwx* represents the corresponding index value in binary. Thus, the selected state indicates both the searched instance value and the index value where the target instance value is found. This second method demonstrates that data already formatted for quantum algorithms requires fewer gates and exhibits greater accuracy.

## Data Availability

All data generated or analysed during this study are included in this published article.
